# More patterns on palliative care identity: An autoethnography contribution

**DOI:** 10.1017/S1478951526102600

**Published:** 2026-05-14

**Authors:** Isabel Galriça Neto, Paula Sapeta, Antonio Almeida, Sofia Meneres

**Affiliations:** 1 Department of Palliative Care, Luz Hospital Lisbon, Lisbon, Portugal; 2 College of Medicine, Lisbon University, Lisbon, Portugal; 3 Catolica Medical School, Portuguese Católica University, Lisbon, Portugal; 4 Health School Lopes Dias, Castelo Branco Institute, Castelo Branco, Portugal; 5 Centro de Investivação Interdisciplinar em Saúde (CIIS)

**Keywords:** Autoethnography, palliative care, interactional patterns, end-of-life care, communication

## Abstract

**Objectives:**

To identify, describe and analytically interpret relational recurrent patterns shaping interactions in PC settings, and to offer practical guidance to haelth care professionals navicating complex end-of-life-scenarios.

**Background:**

This study explores the dynamics influencing relational interactions in palliative care (PC) settings. Building upon 1 author’s extensive clinical experience, reflection, and prior research, we aim to further illuminate the clinical and cultural factors that shape relational interactions and scenarios within PC. By integrating personal observations with scholarly literature and describing specific recurring global patterns of interaction, this article seeks to deepen understanding of PC culture and to provide healthcare professionals with practical strategies to improve engagement with patients and families.

**Methods:**

This study aimed to explore and analytically describe recurrent relational patterns shaping interactions in PC settings through an analytic autoethnographic lens. Short evocative phrases were used to define the identified patterns as clinical vignettes. Based on recurrent clinical observations and reflexive positioning, and through an iterative analytic process, patterns were progressively identified, named, and situated within a theoretical framework. Ethical standards were upheld.

**Results:**

Three end-of-life scenarios – “The Palliative Honeymoon,” “The Cousin of France,” and “Do Everything!” – emerged and were analyzed. The findings emphasize the importance of understanding these behavioral patterns in order to educate health professionals and enhance care provision.

**Significance of results:**

This original Portuguese analytic autoethnographic study is grounded in extensive experiential knowledge and addresses a gap in the literature regarding interactional patterns in PC. By integrating long-standing personal clinical experience with scholarly evidence, this autoethnographic study renders explicit what is often tacit in PC practice – the hidden cultural elements that shape clinical interactions. It is part of a continuum of research that willcontinue and be relate to elements of PC identity. By describing clinically relevant phenomena and integrating them with existing literature, this work offers strong practical implications and contributes to better preparing clinicians for the complex realities of PC practice.

## Introduction

Palliative care (PC) is an evolving field within contemporary healthcare, distinguished not only by its clinical scope but also by its relational, ethical, and cultural complexity. Rather than focusing exclusively on disease-oriented treatment, PC promotes a holistic, person- and family-centered approach that addresses suffering in its multiple dimensions (Radbruch et al. 2020). Within this paradigm, interactions among patients, families, and professionals become central to how care is understood, experienced, and delivered.

The identity of any clinical discipline is shaped not only by its technical practices but also by the recurring ways in which professionals relate to patients, families, colleagues, and knowledge itself (Ibeneme et al. 2017). In PC, these relational configurations emerge through repeated encounters marked by uncertainty, emotional intensity, and the proximity of end-of-life decisions. Such patterns extend beyond communication techniques; they reflect ethical positions, cultural meanings, and shared assumptions about illness, responsibility, and care. Recognizing and articulating these patterns contributes to a deeper understanding of PC as a distinct clinical and cultural field.

Previous literature has described patterns related to illness trajectories, symptom clusters, referral timing, and certain communication phenomena (Stiefel et al. 2024). However, relational interactional patterns as structured, culturally meaningful configurations remain less systematically explored. Existing discussions tend to focus on isolated events or communication strategies rather than on recurring social dynamics that shape expectations, interpretations, and decision-making across time and settings. This gap suggests the need for approaches capable of making visible the tacit knowledge embedded in everyday clinical encounters.

Building on our earlier work on patterns of PC identity (Neto 2022a, 2022b) and drawing on qualitative traditions, this article forms part of a broader project aimed at illuminating the implicit social and cultural logics that inform practice. Using analytic autoethnography (AE) as a methodological framework, guided by Chang’s criteria for analytic AE (Chang 2016), we examine 3 recurrent interactional scenarios commonly observed in end-of-life care: “The Palliative Honeymoon,” “The Cousin of France,” and “Do Everything!” These patterns capture characteristic responses from patients and families in both home and hospital contexts and offer insight into how expectations, emotions, and misunderstandings influence the course of care.

Although analytic AE has gained increasing recognition in health research, particularly in PC research (O’Hara 2018; Grant et al. 2022), the specific relational configurations described here remain largely unarticulated in the literature. By bringing these recurrent dynamics into focus, this study seeks to render explicit dimensions of PC practice that are often experienced but rarely conceptualized. In doing so, it brings knowledge, contributes to a more nuanced understanding of PC culture, and offers practical interpretive tools to support professionals navigating emotionally and ethically complex situations.

## Methods

### Study design

This work follows the methodological orientation of our previous study (Neto 2022b) – cited internationally – adopting a qualitative approach through analytic AE as the primary methodological framework (Chang 2016; O’Hara 2018). According to Chang (2016), five autoethnography standars are defined (Table 1): This approach was selected to explore recurrent relational and complex behavioral patterns observed in PC clinical practice (Grant et al. 2022). Analytic AE is particularly suited to accessing tacit cultural knowledge embedded in everyday clinical interactions and to transforming long-term experiential insight into systematic scholarly analysis (Wall 2008; Arantzamendi et al. 2016; Chang 2016).

**Table 1.** Five standards for autoethnography Table 1 long description.

Standard	Description
Authentic and trustworthy data	Uses authentic data derived from multiple sources, including self-observation, self-reflection, self-analysis, and interviews.
Accountable research process	Clearly describes and critically reflects on the research process.
Ethics toward self and others	Adheres to ethical standards in protecting participants and the autoethnographer; considers the impact of data exposure.
Sociocultural analysis and interpretation	Interprets and explains the sociocultural meanings of human experiences.
Scholarly contribution	Engages meaningfully with existing literature to support broader academic contribution.

Adapted from Chang (2016).

### Researcher positionality

The first author is a senior PC physician with more than 3 decades of continuous clinical experience in both community and hospital settings, including leadership roles in multidisciplinary teams and involvement in national policy development. This prolonged immersion provided sustained access to the social and relational dynamics under study. Recognizing that insider status can both enrich and shape interpretation, reflexive attention was given throughout the research process to the influence of professional identity, authority, and emotional proximity on data construction and analysis.

### Data sources and construction

Data were derived from long-term clinical experience and retrospectively organized into a structured field diary developed for this research. This diary was constructed from personal clinical notes and reflections recorded over time, as well as systematic recollection and documentation of recurrent clinical encounters observed over approximately 30 years of full-time PC practice (in-hospital and community), involving thousands of patients with oncologic and non-oncologic conditions and their families.

Rather than focusing on individual cases, attention was directed toward recurring interactional configurations perceived as culturally and clinically meaningful. Patterns selected for analysis were those repeatedly observed across time and settings, often occurring on a weekly basis, suggesting stability and relevance within PC culture (Mays and Pope 1995; Wall 2008).

All data were anonymized at the time of reconstruction. No identifiable patient information was included.

### Analytic process

Analysis followed an iterative, reflexive process consistent with analytic autoethnographic methodology (Wall 2008; Chang 2016; O’Hara 2018; Grant et al. 2022). The objective was to move from experiential observation to conceptual interpretation of recurring relational patterns. The analytic process included:

- Identification of repeated interactional scenarios through retrospective review of field notes and memory-based reconstructions.
- Reflexive examination of emotional, cultural, and communicative features present in these encounters.
- Comparative analysis across similar situations to detect shared structures, meanings, and triggers.
- Conceptual synthesis of these elements into named patterns serving as heuristic representations of broader sociocultural dynamics.
- Integration of experiential insights with relevant literature to situate interpretations within contemporary theoretical and empirical discussions in PC.

Short evocative labels were developed as analytic devices to synthesize complex constellations of behaviors, beliefs, and emotional responses. These labels function as interpretive constructs rather than anecdotal descriptions.

### Strategies to enhance rigor

Several strategies were employed to enhance methodological rigor:

- Prolonged engagement: Sustained clinical immersion over decades.
- Systematic reflexivity: Ongoing critical reflection on the dual role of clinician and researcher.
- Thick description: Detailed contextualization of relational and cultural dynamics.
- Analytic transparency: Explicit documentation of the pathway from observation to conceptualization.
- Scholarly integration: Iterative engagement with relevant literature.

Rather than aiming for statistical generalizability, the study seeks conceptual transferability through what qualitative scholars describe as “narrative truth” (Mays and Pope 1995; Chang 2016). Informal discussions with 10 experienced colleagues confirmed resonance with their own clinical experiences, further supporting interpretive validity.

### Contextual framing

To situate observed patterns within existing knowledge, a targeted literature search was conducted across major medical and nursing databases (including PubMed, MEDLINE, CINAHL, and Scopus). Relevant empirical and theoretical publications were identified through title and abstract screening and used to contextualize and deepen interpretation during the analytic phase.

### Ethical considerations

This study was considered low risk, as it did not involve direct patient participation or prospective data collection. Ethical principles were nonetheless rigorously upheld. The retrospective field diary excluded any identifiable personal information, and all reconstructed scenarios were presented in a generalized, non-attributable form. Ethical standards and confidentiality procedures were discussed with the local institutional ethics committee.

## Results: Interactional patterns in PC

In analytic AE, findings emerge through the iterative interplay between lived experience, reflexive interpretation, and theoretical integration. The 3 patterns identified here are presented as composite representations of recurrent interactional dynamics observed across multiple clinical encounters over time. The excerpts below are reconstructed from field notes and long-term clinical memory and are intended to illustrate culturally meaningful configurations rather than isolated events.

### The Palliative Honeymoon

This pattern refers to the emotional and interpretive shift that occurs when rapid symptom control leads to visible functional improvement shortly after admission to PC. What initially presents as relief often reveals deeper cultural tensions regarding the meaning of PC. Families frequently interpreted this improvement as evidence that PC has “given life back” and expressed surprise and confusion:

Doctor, this doesn’t make sense… Last week you told us he was very sick. Now he’s eating again and even joking. Are you sure this is still palliative care?

We thought palliative care meant the end. But now he looks better than he did two months ago. Maybe the cancer is improving?

You told us time was short. But now he’s going home. So… was the prognosis wrong?

Patients themselves sometimes articulated similar ambivalence:

I was afraid when you said ‘PC.’ I thought that meant giving up. But I feel more alive now.

Across encounters, these reactions revealed a persistent cultural association between PC and imminent death. When symptom relief contradicted this expectation, families often reinterpreted prognosis, questioned prior medical information, or reconsidered the meaning of referral.

From a reflexive standpoint, I repeatedly noted the emotional intensity of these moments:

I could feel the atmosphere shift from despair to relief. Yet behind the relief was confusion — as if improvement contradicted their narrative of decline.

The “honeymoon” metaphor captures this temporary phase of renewed hope, gratitude, and redefinition of expectations. Ethnographically, it exposes a widespread misunderstanding of PC as synonymous with therapeutic abandonment rather than active, specialized symptom management. The pattern reflects the need for anticipatory communication about the possibility of improvement even in advanced illness.

### The Cousin of France

This scenario describes the arrival of a geographically or emotionally distant relative during advanced stages of illness. The late entrant often brings heightened emotional intensity, moral urgency, and, at times, confrontation.

Typical expressions included:

Why wasn’t I informed earlier? I would never have allowed things to reach this point.

If she had been treated more aggressively, this wouldn’t be happening.

In France, they would have done more.

Are you saying there’s nothing else? That can’t be true.

These interventions frequently destabilized family systems that had reached a fragile equilibrium. Primary caregivers, exhausted from months of involvement, sometimes responded defensively:

We’ve been here every day for months. It’s not that simple.

The emotional charge of these encounters often exceeded the clinical content of the disagreement. In reflexive notes, I observed:

The intensity of his reaction felt disproportionate to the clinical facts, but not to the emotional distance he carried.

Viewed ethnographically, this pattern reflects guilt associated with absence, culturally embedded expectations of filial duty, and the difficulty of confronting finitude from afar. The questioning of prior decisions may represent an attempt to reassert agency, repair perceived absence, or avoid future regret (Table 2). Interestingly, we found an American article that gives another name to the Cousin of France syndrome, “The daughter from California Syndrome” (Unger 2010).

**Table 2.** From field notes to pattern construction: An analytic reflexive pathway (synthesis), the “Palliative Honeymoon” Table 2 long description.

Primary material	Analytic reflexivity	Pattern construction	Practical implications
Narrative extracts (family): “But doctor… how is this possible? He is much better now than a week ago! Yesterday he even ate dessert! The cancer must be improving!” “I thought he was going to die, and now he is going home! Palliative care has given him life back!” “We are in honeymoon!” Patients: very happy, smiling, and expressing gratitude. Personal notes: “They associate palliative care with imminent death, and this improvement contradicts their beliefs.” “I sense an intense emotional climate, with powerful nonverbal cues…” “It is important to explain that palliative care does not shorten life and that good symptom control can improve quality of life.”	I observed similar reactions repeatedly over many years: fear of palliative care, questioning of referral, disbelief followed by relief and happiness – “the honey moon.” I remained aware of my dual role as clinician and observer, and consciously distinguished emotional reactions from analytic interpretation. Literature integration: Palliative care does not shorten survival; early referral is beneficial; and communication strategies are essential.	Elements of the pattern: • Clinical trigger: rapid symptom relief and improved comfort • Cultural belief: palliative care is associated with terminal stages and imminent death • Emotional reactions: confusion, mixed feelings, disbelief, and redefinition of prognosis and hope Emergence of the label: “The Palliative Honeymoon.”	Implications for practice: • Anticipate and openly discuss possible improvements after symptom control • Reframe the role of palliative care to normalize early integration • Promote education and public awareness campaigns among society, policymakers, and healthcare professionals

### Do everything!

The request to “do everything” emerged as one of the most emotionally complex and ethically charged patterns. Although seemingly straightforward, the phrase carried multiple meanings depending on context.

Different formulations illustrated these layers:

Relief-oriented meaning: *“Please do everything so she doesn’t suffer.”*

Hope for life-prolongation*: “Isn’t there at least one more treatment we can try?”*

Symbolic moral imperative: *“We have to fight until the end. That’s what he would want.”*

Avoidance of guilt: *“I don’t want to look back and think we didn’t try hard enough.”*

Technological faith: *“Medicine is so advanced now. There must be something.”*

In more escalated situations: *“If needed, take him to the ICU. We’ll pay for it. Just don’t stop.”*

These requests often placed clinicians in situations of moral tension, particularly when proposed interventions were unlikely to provide benefit. However, reflexive analysis revealed that the phrase rarely functioned as a literal technical directive. Rather, it operated as a cultural script.

As noted in field reflections:

When they say ‘do everything,’ I have learned to pause. The words are simple, but the meanings are layered.

Ethnographically, this pattern reflects deeply rooted beliefs about medical omnipotence, moral responsibility, and the symbolic necessity of “fighting” death. It also expresses anticipatory grief and fear of abandonment. The tension lies not between action and inaction, but between technological intervention and proportionate care.

Recognizing the multiplicity embedded within the phrase enables clinicians to move from reactive defensiveness to exploratory dialogue – clarifying goals, values, and understanding of prognosis before discussing specific interventions (Quill et al. 2009; Karlin et al. 2024).

## Discussion

This study extends previous work by offering an analytic autoethnographic exploration of culturally embedded relational patterns that shape PC practice and identity. By drawing on long-term clinical immersion and reflexive analysis, it brings forward forms of tacit knowledge that often remain implicit in everyday interactions among patients, families, and professionals. The 3 patterns described here can be understood as culturally mediated responses to serious illness and end-of-life transitions, reflecting broader social narratives about medicine, suffering, responsibility, and death.

### The “Palliative Honeymoon”

The “Palliative Honeymoon” illustrates a persistent gap between public perceptions of PC and its clinical reality. When symptom relief leads to visible functional improvement, families may interpret this as evidence that the disease is less advanced than previously thought or that referral to PC was premature. These reactions reveal the durability of a widespread belief that PC is synonymous with imminent death or therapeutic abandonment (Roy 2012; Dixe et al. 2020; Flieger et al. 2020; Zimmermann et al. 2021).

This pattern highlights how misunderstandings about the scope and timing of PC continue to shape expectations and emotional responses (Temel et al. 2010; Strand et al. 2013; Zimmermann and Mathews 2022; European Association for Palliative Care 2024; Petrillo et al. 2024). Rather than signaling the end of active treatment, PC often produces meaningful improvements in comfort, function, and quality of life (Temel et al. 2010; Zimmermann and Mathews 2022; Masters et al. 2024; Petrillo et al. 2024). The surprise experienced by patients and families when this occurs underscores the need for proactive communication and public education. Clinicians may benefit from anticipating these reactions, explicitly addressing common misconceptions, and framing PC as an active, supportive approach that can coexist with ongoing treatment (Gomes 2015; Petrillo et al. 2024) (Tables 2 and 3).

**Table 3.** Top 10 messaging principles that palliative care clinicians should know for public communication Table 3 long description.

Tip	Recommendation
1	Remember that public messaging introduces people to a specialized form of medical care; do not assume prior knowledge of palliative care.
2	Define your target audience and tailor messages to what matters to them, not to professional priorities.
3	Emphasize the benefits of palliative care rather than its technical features.
4	Use aspirational images that portray living well with serious illness.
5	Frame palliative care as supporting quality of life while living with serious illness.
6	Use stories that illustrate palliative care done well; do not rely solely on statistics.
7	Anchor messages in clear facts and lead with the key idea you want audiences to remember.
8	Deliver messages through voices and perspectives from diverse communities.
9	Use simple, conversational language and avoid excessive medical terminology.
10	When developing and evaluating messaging, include people from the intended audience.

Adapted from Masters et al. (2024) and Back et al. (2015).

More broadly, this scenario reflects how deeply embedded cultural narratives influence the interpretation of clinical events. The temporary restoration of well-being can disrupt established assumptions about illness trajectory and prognosis, prompting a renegotiation of meaning around the patient’s condition. Recognizing this dynamic may help clinicians support families in integrating new information without generating confusion or false hope (Back et al. 2024). When the evidence points to an increase in global burden of suffering in health care (Sleeman et al. 2019; Kwete et al. 2024), it becomes more vital to increase society’s literacy about PC and its benefits, including the information that PC is a true Human Right (Brennan 2007; Breitbart 2008), which remains unfulfilled.

### The “Cousin of France”

This dynamic was described under the name of “The daughter from Califórnia” in the American context, with small cultural differences (Unger 2010). Then, in Spain, 2 authors (Sancho 2005; Bermejo 2019) were among the first who wrote about this scenario in Europe, under the name “Son of Bilbao.” Twenty-five years ago, in Portugal, we adapted it as the aforementioned “Cousin of France Syndrome.”

That pattern captures the complex relational and emotional dynamics that emerge when a previously distant relative reenters the family system during advanced illness. These encounters are often marked by disbelief, questioning of prior decisions, and, at times, open conflict (Kristjanson and Aoun 2004; Farabelli et al. 2020). Rather than viewing such reactions solely as disruptive or interpersonal conflict, an ethnographic perspective allows them to be understood as expressions of guilt, grief, moral responsibility, and the difficulty of confronting impending loss from a distance.

The late arrival of a relative may destabilize a fragile equilibrium that has developed among those who have been directly involved in caregiving. Challenges to established treatment plans can reflect attempts to reassert agency, repair perceived absence, or make sense of a situation that feels sudden and overwhelming. In this sense, the pattern is less about individual personalities and more about broader cultural expectations surrounding kinship, duty, and protection (Kristjanson and Aoun 2004; Bermejo 2019; Farabelli et al. 2020; Flugelman 2021). Recognizing this configuration allows clinicians to anticipate escalation, structure family meetings proactively, and validate emotions without undermining established care plans.

From a clinical perspective, these situations call for structured and compassionate communication. Active listening, validation of emotions, and clarification of the clinical context can help reduce confrontation and facilitate shared understanding. Family meetings may provide a space for alignment (Omilion-Hodges and Swords 2017; Widera et al. 2020; Flugelman 2021), allowing previously uninvolved relatives to become integrated into the decision-making process without undermining those who have carried the burden of care (Table 4). Supporting the need to say goodbye and to close cycles is very important (Bujdos et al. 2024). Recognizing the underlying grief and anticipatory mourning that often drive these reactions may also help clinicians respond with empathy rather than defensiveness (Kristjanson and Aoun 2004; Farabelli et al. 2020).

**Table 4.** Summary: Managing the “Cousin of France” pattern Table 4 long description.

Emotions involved	Suggested professional strategies
Incredulity and difficulty accepting reality	Listen actively to concerns and assess understanding of the patient’s condition.
Guilt (whether justified or not)	Acknowledge and validate emotions without judgment; reassure that affection and concern are recognized.
Anger and hostility	Avoid confrontation; validate emotions while maintaining respectful boundaries.
Pain of loss (anticipatory grief)	Show empathy, reflect feelings, and encourage open communication about unresolved concerns.
Family conflict and accusations	Facilitate family meetings to clarify the clinical situation, align expectations, and promote mutual understanding.

### Do everything!

Requests to “Do everything” represent one of the most emotionally charged and ethically complex moments in PC practice. They encapsulate tensions between technological imaginaries of medicine and the inevitability of death (Quill et al. 2009; Yim et al. 2022; Awdish et al. 2024; Karlin et al. 2024). Although the phrase appears to be a clear directive, its meaning is rarely straightforward. It may express a desire to relieve suffering, a hope for life-prolonging treatment, a moral obligation to fight for survival, or an attempt to cope with the impending loss of a loved one.

Viewed through an ethnographic lens, this pattern reflects a broader cultural script shaped by faith in medical technology, social expectations about perseverance, and the symbolic importance of not giving up. For many families, the request is less about specific interventions and more about ensuring that nothing essential is left undone. It may represent an effort to protect themselves from future regret or to demonstrate care and loyalty.

For clinicians, these moments can generate moral tension, particularly when proposed interventions are unlikely to provide benefit or may increase suffering (Meier et al. 2001; Back et al. 2015). Responding effectively requires moving beyond the literal wording of the request to explore its underlying meanings. Clarifying goals, values, and understanding of prognosis can help reframe decisions around comfort, dignity, and proportionality of care (Table 5). In this process, the focus shifts from doing “everything” technologically possible to doing everything that is clinically and ethically appropriate and aligned with the patient’s best interests (Quill and Cassel 1995; Clark 2002; Chochinov 2023). The principle of adequacy – rather than limitation – of therapeutic effort better captures the moral imperative to provide intensive caring, as Chochinov named it, focused on comfort, dignity, avoiding futility (Taylor and Lightbody 2018) and non-abandonment (Quill and Cassel 1995^)^. In any case, we are obliged to be technically competent and committed to humanized practice (Council of Europe 2014; Flugelman 2023).

**Table 5.** Different treatment perspectives underlying requests to “Do everything” Table 5 long description.

Possible meaning of the request
Everything that might provide maximum relief of suffering, even if it might unintentionally shorten life.
Everything that has a reasonable chance of prolonging life, but not if it would significantly increase suffering.
Everything that has a reasonable chance of prolonging life, even if it may cause a modest increase in suffering.
Everything that has a reasonable chance of prolonging life, even for a short time, regardless of its effect on suffering.
Everything that has any possible potential to prolong life, even minimally, regardless of its impact on suffering.

Adapted from Quill et al. (2009), Karlin et al. (2024), and Yim et al. (2022).

Across the 3 patterns, a shared cultural thread becomes visible: difficulty integrating finitude into contemporary medical and social consciousness. Each scenario represents a point of friction between deeply held narratives – about hope, duty, control, and technological progress – and the lived reality of serious illness.

These interactions are not merely individual events but expressions of broader social meanings that shape how patients and families interpret illness and respond to care. The patterns described here function as interpretive tools rather than diagnostic categories. The findings also illustrate the value of ethnographic approaches in capturing dimensions of care that are not easily accessible through quantitative methods (Mathieu and Hagelsteen 2026). By rendering these dynamics explicit, analytic AE transforms tacit experiential knowledge into transferable clinical insight.

PC, in this context, represents not a withdrawal of medicine but an evolution toward a more comprehensive response to suffering. The patterns described here highlight how misconceptions, emotional complexity, and relational tensions can influence decision-making and communication. Making these dynamics visible allows clinicians to better anticipate challenges and respond with greater sensitivity and clarity.

A central strength of this study lies in its grounding in prolonged clinical immersion, which enabled the identification of recurring interactional configurations over time. The analytic autoethnographic approach allowed for the integration of experiential knowledge with scholarly reflection, generating interpretations that are closely connected to practice. The patterns described are intended as conceptual frameworks that may resonate with clinicians who recognize similar dynamics in their own settings.

This study has limitations inherent to analytic AE, as interpretations are shaped by the researcher’s positionality, professional background, and retrospective memory. Although reflexivity was maintained throughout, the findings are not statistically representative and may vary across cultural and organizational contexts. As part of an ongoing research program, future work will incorporate cross-national clinician interviews to triangulate and further refine these patterns, strengthening their theoretical contribution.

Despite these limitations, the study underscores the importance of attending to relational and cultural dimensions of care. Training in PC often emphasizes symptom control and clinical decision-making, yet communication, meaning-making, and family dynamics are equally central to practice. By naming and describing recurring patterns, this work aims to support clinicians in recognizing complex situations, anticipating emotional responses, and engaging more effectively with patients and families.

In this way, analytic AE contributes to a deeper understanding of the human context in which PC unfolds. As the field continues to evolve globally, integrating such perspectives may help foster more reflective, compassionate, and culturally responsive care.

This study reveals socio-culturally patterned behaviors that shape interactions in PC, contributing to a deeper understanding of its culture and identity. Building on previous work and using a coherent analytic autoethnographic approach, it offers new perspectives on familiar clinical scenarios. By providing practical interpretive tools that support compassionate communication and better alignment between patient values, family needs, and professional responsibilities, the study holds clear clinical relevance. As PC expands globally, integrating ethnographic insight into practice may help foster more humanized, equitable, and culturally responsive care.

## Table 1 Long description

The table lists five standards used to evaluate autoethnographic work and briefly defines each one. “Authentic and trustworthy data” emphasizes using genuine evidence from multiple sources such as self-observation, reflection, analysis, and interviews. “Accountable research process” focuses on clearly describing the methods and critically reflecting on how the study was conducted. “Ethics toward self and others” highlights protecting participants and the researcher and considering risks from exposing data. “Sociocultural analysis and interpretation” requires explaining the broader cultural and social meanings of lived experiences. “Scholarly contribution” stresses engaging with existing literature so the work contributes to academic knowledge. The entries are qualitative definitions rather than ranked or quantified measures, so no numerical comparisons or trends are provided.

Navigate back to Table 1.

## Table 2 Long description

The table links qualitative field notes to an analytic pattern and practice actions around families’ reactions after palliative care referral. Narrative quotes describe surprise and disbelief when symptoms improve quickly (e.g., “the cancer must be improving,” “palliative care has given him life back,” “we are in honeymoon”), alongside observations of patients appearing happy and grateful. Analytic reflexivity notes the clinician-researcher’s dual role and separating emotional responses from interpretation, then integrates literature that early palliative care is beneficial and does not shorten survival. The constructed pattern identifies triggers and components: rapid symptom relief, a cultural belief that palliative care equals imminent death, and emotional responses such as confusion, mixed feelings, and redefinition of prognosis and hope, culminating in the label “The Palliative Honeymoon.” Practical implications emphasize anticipating and discussing possible improvement after symptom control, reframing palliative care as appropriate earlier in illness, and promoting education and public awareness among the public, policymakers, and healthcare professionals. The content is interpretive and based on repeated observations and selected extracts rather than quantitative frequencies.

Navigate back to Table 2.

## Table 3 Long description

The table lists 10 numbered recommendations for how clinicians should communicate about palliative care to the public. It emphasizes that public audiences may not know what palliative care is, so messages should avoid assumptions and use simple, conversational language with minimal medical jargon. Several tips focus on audience fit: define the target audience, prioritize what matters to them, and include intended audience members when developing and evaluating messages. The recommendations favor benefit- and quality-of-life framing—highlighting how palliative care supports living well with serious illness—rather than technical features. Communication approaches include using aspirational images, telling stories that show palliative care done well instead of relying only on statistics, and leading with a clear key idea grounded in facts. The table also calls for delivering messages through voices and perspectives from diverse communities. These are qualitative principles rather than measured outcomes, so the table does not rank effectiveness or provide performance data.

Navigate back to Table 3.

## Table 4 Long description

The table links five emotional reactions from family members to recommended professional strategies for responding. Incredulity and difficulty accepting reality are addressed by active listening and checking the person’s understanding of the patient’s condition. Guilt is met with nonjudgmental acknowledgment and reassurance that care and concern are recognized. Anger and hostility call for avoiding confrontation while validating feelings and maintaining respectful boundaries. Anticipatory grief is supported through empathy, reflecting feelings, and encouraging open discussion of unresolved concerns. Family conflict and accusations are managed by facilitating family meetings to clarify the clinical situation, align expectations, and promote mutual understanding.

Navigate back to Table 4.

## Table 5 Long description

The table lists possible interpretations of a request to “do everything” in medical care. One meaning emphasizes maximum relief of suffering, even if treatment could unintentionally shorten life. The remaining meanings focus on life prolongation with progressively fewer limits based on suffering: from pursuing treatments with a reasonable chance of prolonging life only if they do not significantly increase suffering, to accepting a modest increase in suffering. More aggressive interpretations include prolonging life even briefly regardless of suffering, and finally using any intervention with any possible potential to extend life, even minimally, regardless of impact on suffering. Overall, the entries form a continuum from comfort-focused goals to increasingly maximal life-prolonging goals. The table provides qualitative categories rather than frequencies, so it does not indicate how common each meaning is.

Navigate back to Table 5.

## References

[ref1] Arantzamendi M, Lopez-Dicastillo O, Robinson C, et al. (2016) Investigación cualitativa en cuidados paliativos: un recorrido por los enfoques más habituales. *Medicina Paliativa* 24, 219–226.

[ref2] Awdish RLA, Grafton G and Berry LL (2024) Never-words: What not to say to patients with serious illness. *Mayo Clinic Proceedings* 99(10), 1553–1557. doi:10.1016/jmayocp.2024.06.003. 39177542

[ref3] Back AL, Rotella JD, Dashti A, et al. (2024) Top 10 tips palliative care clinicians should know about messaging for the public. *Journal of Palliative Medicine* 27(3), 405–410. doi:10.1089/jpm.2023.0405. 37738320 PMC11074435

[ref4] Back AL, Rushton CH, Kaszniak AW, et al. (2015) “Why are we doing this?” Clinician helplessness in the face of suffering. *Journal of Palliative Medicine* 18(1), 26–30. doi:10.1089/jpm.2014.0202. 25555085

[ref5] Bermejo JC (2019) Counseling at the end of life and in the grief. *Revista Clinica Contemporanea* 10, e2.

[ref6] Breitbart W (2008) Palliative care as a human right. *Palliative and Supportive Care* 6(4), 323–325. doi:10.1017/S1478951508000504. 19006585

[ref7] Brennan F (2007) Palliative care as an international human right. *Journal of Pain and Symptom Management* 33(5), 494–499. doi:10.1016/j.jpainsymman.2006.10.23. 17482036

[ref8] Bujdos V, Chekan K, Marterre B, et al. (2024) The last visit: Saying goodbye to patients. *Ournal of Palliative Medicine* 27(10), e71–2. doi:10.1089/jpm.2024.0191. PMC11807905 39469767

[ref9] Chang H (2016) Autoethnography in health research: Growing pains? *Qualitative Health Research* 26(4), 443–451. doi:10.1177/1049732315619386. 26880757

[ref10] Chochinov HM (2023) Intensive caring: Reminding patients they matter. *Journal of Clinical Oncology* 41(16), 2884–2887. doi:10.1200/JCO.23.00620. 37075272 PMC10414729

[ref11] Clark D (2002) Between hope and acceptance: The medicalization of dying. *BMJ* 324(7342), 905–907. doi:10.1136/bmj.324.7342.905. 11950744 PMC1122840

[ref12] Council of Europe (2014) *Guide on the Decision-making Process regarding Medical Treatment in End-of-life Situations*. Strasbourg: Council of Europe.

[ref13] Dixe MA, Santo IDO, Lopes S, et al. (2020) Knowledge and myths about palliative care among the general public and health care professionals in Portugal. *International Journal of Environmental Research & Public Health* 17(13), 4630. doi:10.3390/ijerph17134630. 32605086 PMC7369792

[ref14] European Association for Palliative Care (2024) White paper on standards and norms for hospice and palliative care in Europe: Part 1. *European Journal of Palliative Care* 31(2), 1–20.

[ref15] Farabelli JP, Kimberly SM, Altilio T, et al. (2020) Top ten tips palliative care clinicians should know about psychosocial and family support. *Journal of Palliative Medicine* 23(2), 280–286. doi:10.1089/jpm.2019.0200. 31687876

[ref16] Flieger SP, Chui K and Koch-Weser S (2020) Lack of awareness and common misconceptions about palliative care among adults: Insights from a national survey. *Journal of General Internal Medicine* 35, 2059–2064. doi:10.1007/s11606-020-05803-0. 32157652 PMC7351936

[ref17] Flugelman MY (2021) How to talk with the family of a dying patient. *BMJ Supportive & Palliative Care* 11(4), 418–421. doi:10.1136/bmjspcare-2020-002743. PMC8606451 34312184

[ref18] Flugelman MY (2023) A guide for caring with dignity and compassion at the end of life. *Ournal of Palliative Medicine* 26(3), 123–125. doi:10.1089/jpm.2022.0405.

[ref19] Gomes B (2015) Palliative care: If it makes a difference, why wait? *Journal of Clinical Oncology* 33(13), 1420–1421. doi:10.1200/JCO.2014.60.1412. 25800757

[ref20] Grant MP, Philip JAM, Deliens L, et al. (2022) Understanding complexity in care: Opportunities for ethnographic research in palliative care. *Journal of Palliative Care* 38(1), 1–8. doi:10.1177/08258597211072336. 35167402

[ref21] Ibeneme S, Eni G, Ezuma A, et al. (2017) Roads to health in developing countries: Understanding the intersection of culture and healing. *Current Therapeutic Research-clinical and Experimental* 84, 1–6. doi:10.1016/j.curtheres.2017.01.001. 29234482 PMC5717292

[ref22] Karlin D, Pham C, Furukawa D, et al. (2024) Use of antimicrobials at the end of life: A state-of-the-art review. *Clinical Infectious Diseases* 78(3), e27–36. doi:10.1093/cid/ciad657. 38301076

[ref23] Kristjanson LJ and Aoun S (2004) Palliative care for families: Remembering the hidden patients. *The Canadian Journal of Psychiatry* 49(6), 359–365. doi:10.1177/070674370404900603. 15283530

[ref24] Kwete XJ, Bhadelia A, Arreola-Ornelas H, et al. (2024) Global assessment of palliative care need: Serious health-related suffering measurement methodology. *Journal of Pain and Symptom Management* 68(2), e116–37. doi:10.1016/j.jpainsymman.2023.10.006. 38636816 PMC11253038

[ref25] Masters JL, Josh PW, Kirkpatrick AJ, et al. (2024) Providing clarity: communicating the benefits of palliative care beyond end-of-life support. *Palliative Care and Social Practice* 18, 26323524241263109. doi:10.1177/26323524241263109. 39045294 PMC11265247

[ref26] Mathieu C and Hagelsteen K (2026) Enhancing reflexivity in research and practice in healthcare through oral-based autoethnography. *Qualitative Health Research* 36(1), 15–29. doi:10.1177/10497323241234567. 39874420 PMC12675833

[ref27] Mays N and Pope C (1995) Rigour and qualitative research. *BMJ* 311(6997), 109–112. doi:10.1136/bmj.311.6997.109. 7613363 PMC2550154

[ref28] Meier DE, Back AL and Morrison RS (2001) The inner life of physicians and care of the seriously ill. *JAMA* 286(23), 3007–3014. doi:10.1001/jama.286.23.3007. 11743845

[ref29] Neto IG (2022a) *Da Ciência, Do Amor E Do Valor da Vida*. Lisboa: Oficina do Livro.

[ref30] Neto IG (2022b) Palliative care and its own identity, through an autoethnography: Do you recognize these patterns? *Palliative Care and Social Practice* 16, 26323524221122346. doi:10.1177/26323524221122346. 36118620 PMC9478704

[ref31] O’Hara S (2018) Autoethnography: The science of writing your lived experience. *HERD: Health Environments Research & Design Journal* 11(4), 14–17. doi:10.1177/1937586717739386. 30336695

[ref32] Omilion-Hodges LM and Swords NM (2017) Communication matters: Exploring the intersection of family and practitioner end of life communication. *Behavioral Sciences* 7(1), 15. doi:10.3390/bs7010015. 28335501 PMC5371759

[ref33] Petrillo LA, Jones KF, El-Jawahri A, et al. (2024) Why and how to integrate early palliative care into cutting-edge personalized cancer care. *American Society of Clinical Oncology Education Book* 44, e100038. doi:10.1200/EDBK_100038. 38815187

[ref34] Quill TE, Arnold R and Back AL (2009) Discussing treatment preferences with patients who want “everything.” *Annals of Internal Medicine* 151(5), 345–349. doi:10.7326/0003-4819-151-5-200909010-00010. 19721022

[ref35] Quill TE and Cassel CK (1995) Nonabandonment: A central obligation for physicians. *Annals of Internal Medicine* 122, 368–374. doi:10.7326/0003-4819-122-5-199503010-00008. 7847649

[ref36] Radbruch L, De Lima L, Knaul F, et al. (2020) Redefining palliative care: A new consensus-based definition. *Journal of Pain and Symptom Management* 60(4), 754–764. doi:10.1016/j.jpainsymman.2020.04.027. 32387576 PMC8096724

[ref37] Roy DJ (2012) Untruths about palliative care. *Journal of Palliative Care* 28(1), 3–4. 22582465

[ref38] Sancho MG (2005) *To Die with Dignity*. Madrid: Aran Ediciones.

[ref39] Sleeman KE, De Lima L, Radbruch L, et al. (2019) The escalating global burden of serious health-related suffering. *The Lancet Global Health* 7(7), e883–92. doi:10.1016/S2214-109X(19)30172-X. 31129125 PMC6560023

[ref40] Stiefel F, Saraga M and Bourquin C (2024) Collusion revisited: Polyadic collusions and their contextual determinants. *Journal of Contemporary Psychotherapy* 54, 19–27. doi:10.1007/s10879-023-09617-4.

[ref41] Strand JJ, Kamdar MM and Carey EC (2013) Top 10 things palliative care clinicians wished everyone knew about palliative care. *Mayo Clinic Proceedings* 88(8), 859–865. doi:10.1016/j.mayocp.2013.04.013. 23910412

[ref42] Taylor DR and Lightbody CJ (2018) Futility and appropriateness: Challenging words, important concepts. *Postgraduate Medical Journal* 94(1110), 238–243. doi:10.1136/postgradmedj-2017-135323. 29477988

[ref43] Temel JS, Greer JA, Muzikansky A, et al. (2010) Early palliative care for patients with metastatic non-small-cell lung cancer. *New England Journal of Medicine* 363(8), 733–742. doi:10.1056/NEJMoa1000678. 20818875

[ref44] Unger KM (2010) The Daughter from California syndrome. *Journal of Palliative Medicine* 13(12), 1405. doi:10.1089/jpm.2010.0340. 21155638

[ref45] Wall S (2008) Easier said than done: Writing an autoethnography. *International Journal of Qualitative Methods* 7(1), 38–53. doi:10.1177/160940690800700103.

[ref46] Widera E, Anderson WG, Santhosh L, et al. (2020) Family meetings on behalf of patients with serious illness. *New England Journal of Medicine* 383(11), e71. doi:10.1056/NEJMvcm2013414. 32905678

[ref47] Yim H, Hashmi SS, Dewar B, et al. (2022) Everything has been tried and his heart can’t recover…. *BMC Medical Ethics* 23(1), 66. doi:10.1186/s12910-022-00823-3. 35761229 PMC9237977

[ref48] Zimmermann C and Mathews J (2022) Palliative care is the umbrella, not the rain—a metaphor to guide conversations in advanced cancer. *JAMA Oncology* 8(5), 681–682. doi:10.1001/jamaoncol.2012.6748. 35297961

[ref49] Zimmermann C, Wong JL, Swami N, et al. (2021) Public knowledge and attitudes concerning palliative care. *BMJ Support Palliat Care* 11, 1–8. doi:10.1136/bmjspcare-2020-002743. 34620693

